# Cone Beam Computed Tomography (CBCT) Evaluation of Alveolar Bone Thickness and Root Angulation in Anterior Maxilla for Planning Immediate Implant Placement

**DOI:** 10.7759/cureus.37875

**Published:** 2023-04-20

**Authors:** Rutvi Vyas, Sonam Khurana, Dhruv Khurana, Steven RRRRR. Singer, Adriana G Creanga

**Affiliations:** 1 Oral and Maxillofacial Radiology, University of Florida Health, Gainesville, USA; 2 Oral Pathology, Radiology, and Medicine, New York University (NYU) College of Dentistry, New York, USA; 3 Addiction Psychiatry, University of California Los Angeles David Geffen School of Medicine, Los Angeles, USA; 4 Diagnsotic Sciences, Rutgers, Newark, USA; 5 Diagnostic Sciences, Rutgers School of Dental Medicine, Newark, USA; 6 Oral and Maxillofacial Radiology, Rutgers School of Dental Medicine, Newark, USA

**Keywords:** cortical thickness, root angulation, immediate implants, anterior maxilla, cone- beam computed tomography

## Abstract

Objective: This retrospective study aimed to measure the labial, palatal, mesial, and distal bone thickness around maxillary central and lateral incisors and canines and height from crest to apex, using cone beam computed tomography (CBCT) images and compare the results based on gender. The second objective of the study was to measure root angulation on CBCT images and its relation with the labial cortical thicknesses.

Material and Methods: After the Institutional Review Board (IRB) approval, a total of 140 CBCT volumes were included in this study according to the set criteria. On each scan, right-side maxillary central, lateral incisors, and canine were selected for the measurements. All the measurements were done at three levels at the alveolar crest (L1), mid-root (L2), and apical region (L3) for each tooth.

Results: The Student’s t-test was performed to compare the result of buccal, palatal, mesial, and distal bone thickness, angulation, and height of all subjects. Buccal alveolar bone thickness was minimum at the mid-root region, and the palatal bone thickness was minimum at the crestal region. The mesial bone thickness was minimum at the mid-root level, and distal bone thickness was minimum at the crest level. The available bone height was maximum at the lateral incisor and equal for the central incisor and canine. The canine was the most angulated tooth.

Conclusion: Cone beam computed tomography is a reliable imaging modality to evaluate pre-surgical immediate implant sites and measure alveolar bone thickness. The canine was the most angulated tooth with more buccal alveolar bone thickness.

## Introduction

Adequate buccal bone support is necessary for the primary and long-term stability of dental implants. Sufficient bone thickness is essential to support the implant circumferentially. Immediate placement of implants has a long history since the 1980s and has been evolving as a reliable treatment option for any failed tooth [1]. Immediate implants have demonstrated several benefits to a patient suffering from a failed or a diseased tooth problem. Implants immediately replace the missing tooth, shortening the toothless phase and affecting esthetics. They can provide a quicker solution to the failed tooth while preserving the tissue architecture, further enhancing implant stability [2]. The anterior maxilla is the prime esthetic zone of the mouth and the most common site prone to injury and trauma amongst the young population worldwide. The demand for highly esthetic restorations in this area is increasing and implant is the restoration of choice [3-4]. It is essential to have a conservative approach while achieving an excellent esthetic replacement [5]. Several aspects need to be considered while planning immediate implant placement using cone beam computed tomography (CBCT), including the angulation of the root, the thickness of the surrounding alveolar bone, the proximity of nearby anatomical structures, and the bone architecture around the implant, which might have implications for the long-term success of the implant [6]. CBCT is the recommended method for preoperative evaluation of implant sites [7]. This retrospective study aimed to measure the labial, palatal, mesial, and distal bone thickness around maxillary central and lateral incisors and canines and height from crest to apex, using CBCT and compare the results based on gender. The second objective of the study was to measure root angulation on CBCT images and its relation with the labial cortical thicknesses. These objectives are considered for more detailed planning of the immediate implant placements in the anterior maxillary region.

## Materials and methods

The retrospective study was performed in the diagnostic science department, with the approval of the Institutional Review Board (IRB no. Pro20170001788). The limited field CBCTs were extracted from the database of dental schools. All the CBCT volumes were already acquired for some other purpose on iCAT (Imaging Science International, Hatfield, PA) using 18 mA, 110 kV, a scanning time of 8.9 s, and image acquisition at 0.3-mm voxel size. The CBCT volumes were viewed as multiplanar reformatted images at a reconstructed slice thickness of 1 mm. The inclusion criteria for the study were: 1) scans that demonstrated the six maxillary anterior teeth from canine to canine; 2) The patient's age should be greater than 18, irrespective of gender. The exclusion criteria for the study were: 1) The presence of any tooth or bone anomalies/pathologies in the maxillary anterior region; 2) Trauma to anterior maxillary teeth, which could affect the maxillary anterior region; and 3) Any CBCT volumes that are not of diagnostic quality due to implants and metallic restorations in or near the anterior maxilla and mandible.

A total number of 940 CBCT volumes were examined by two investigators taken between February 2010 and December 2017. According to the set criteria, 140 CBCT volumes were included in this study. All the CBCT volumes were de‐identified before any measurements, and information about gender was preserved. Among 140, 96 female and 44 male scans were selected. On each scan, the right-side maxillary central, lateral incisors, and canine were chosen for the measurements.

Measurements

**Figure 1:**
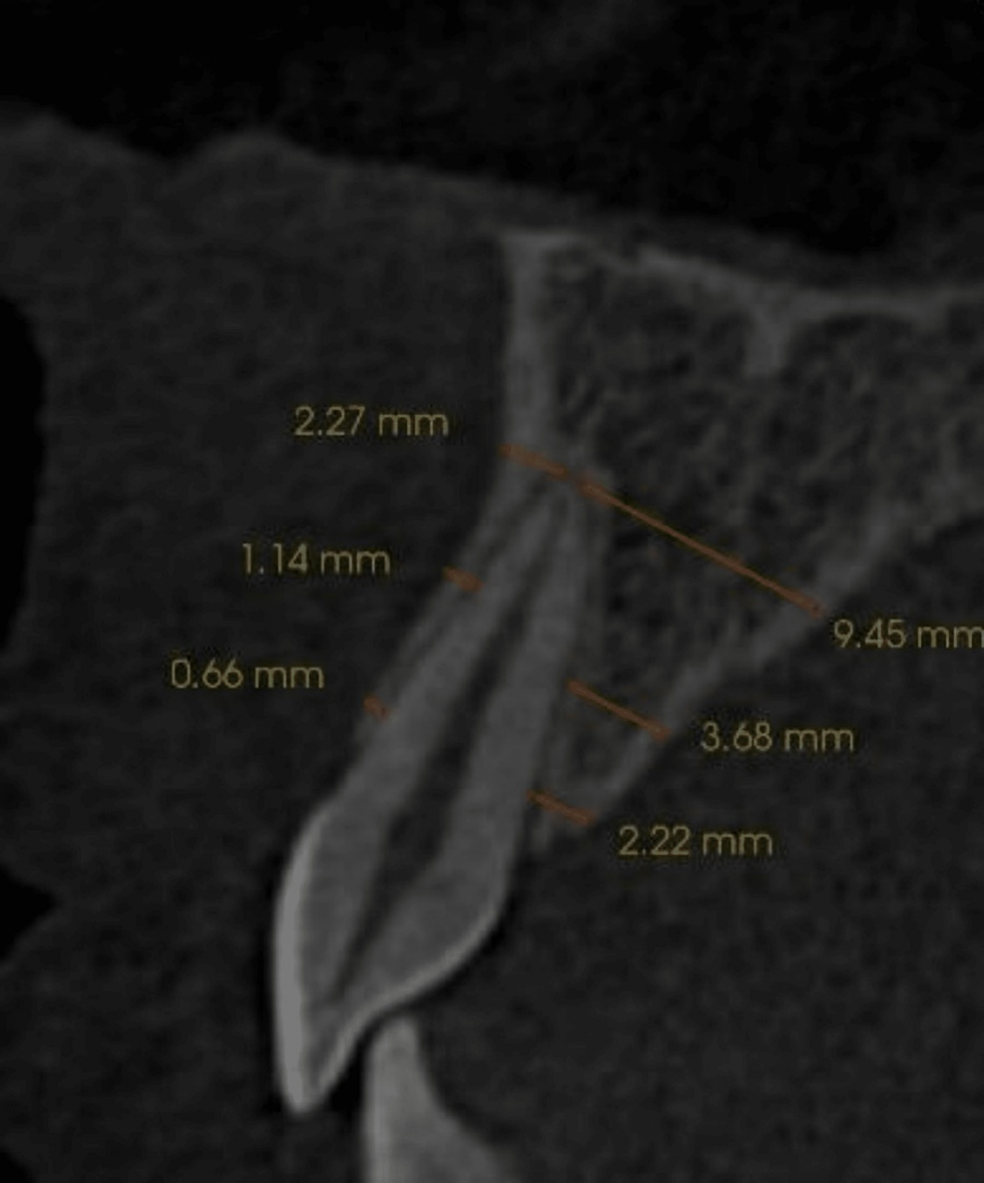
The orange lines at L1 , L2, L3 depict width of labial and palatal cortex.

After collecting all the data, all the images were saved in Digital Imaging and Communications in Medicine (DICOM) format, and measurements were done in InVivo Dental imaging software (version 5.4.4, Anatomage, San Jose, CA). Before measuring, all the images were realigned parallel to Frankfort‐horizontal (FH) plane in the sagittal plane. A curved arch reconstruction was done before measurements to decide the three levels at the alveolar crest (L1), mid-root (L2), and apical region (L3) for each tooth. The bone width was measured in labial, palatal, mesial, and distal directions. The labial width was measured from the labial cortex to the labial surface of the teeth. The palatal width was calculated from the palatal surface of the tooth to the palatal cortex (Figure 1).

**Figure 2:**
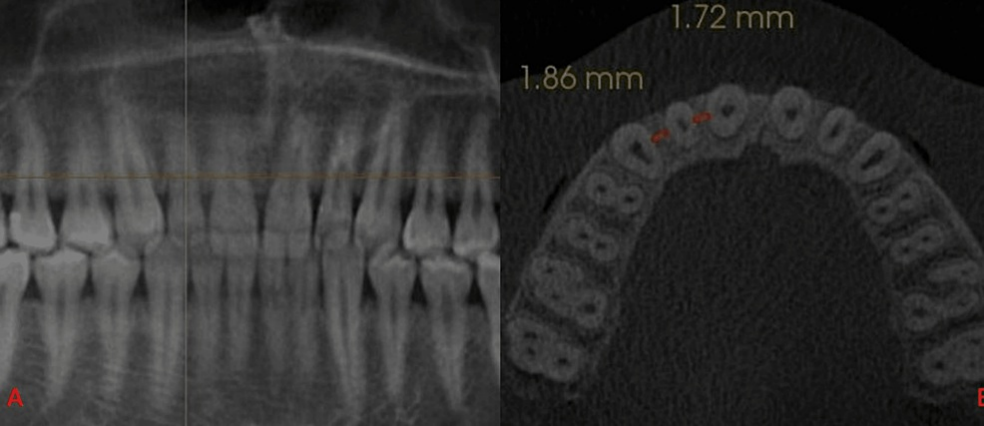
(A) Horizontal orange line depicts L1. (B) Orange lines mesial and distal to lateral incisor depict the bone width at L1.

**Figure 3:**
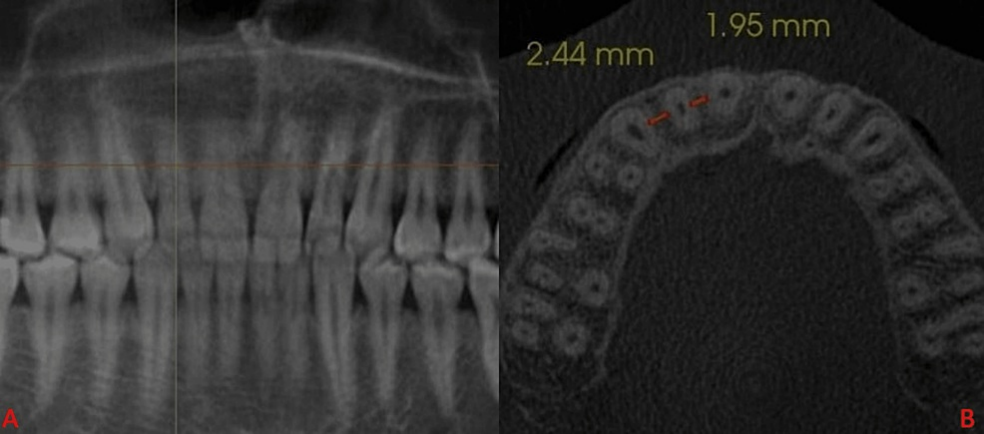
(A) Horizontal orange line depicts L2. (B) Orange lines mesial and distal to lateral incisor depict the bone width at L2.

**Figure 4:**
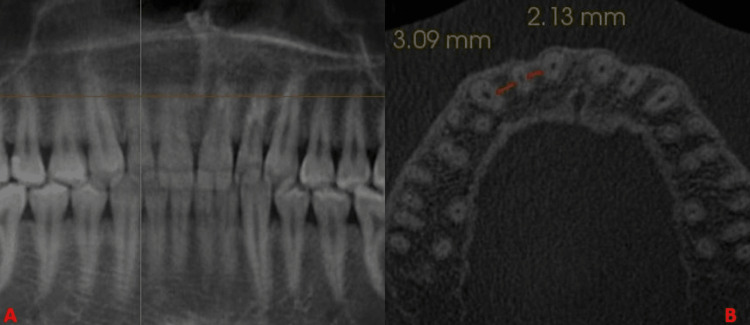
(A) Horizontal orange line depicts L3. (B) Orange lines mesial and distal to lateral incisor depict the bone width at L3.

The mesial and distal widths were measured between mesial and distal surfaces of adjacent teeth at L1, L2, and L3 levels (Figures 2-4).

 

**Figure 5:**
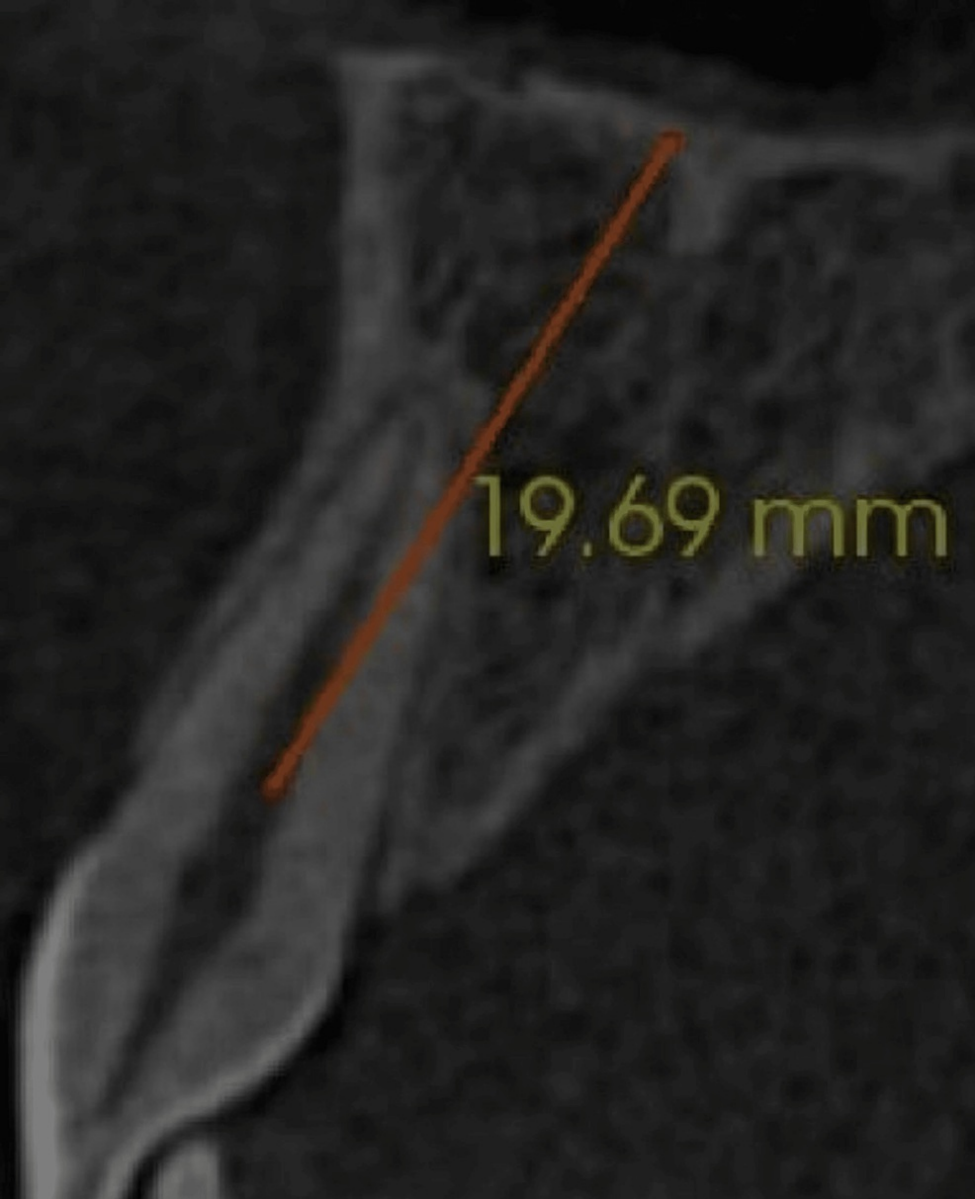
Orange line depicts bone height measured from the center of the tooth at the alveolar crest level to the floor of the nose.

 The bone height was measured on a cross-section by a vertical line drawn at the center of the tooth at the alveolar crest level to the nose floor (Figure 5). 

**Figure 6:**
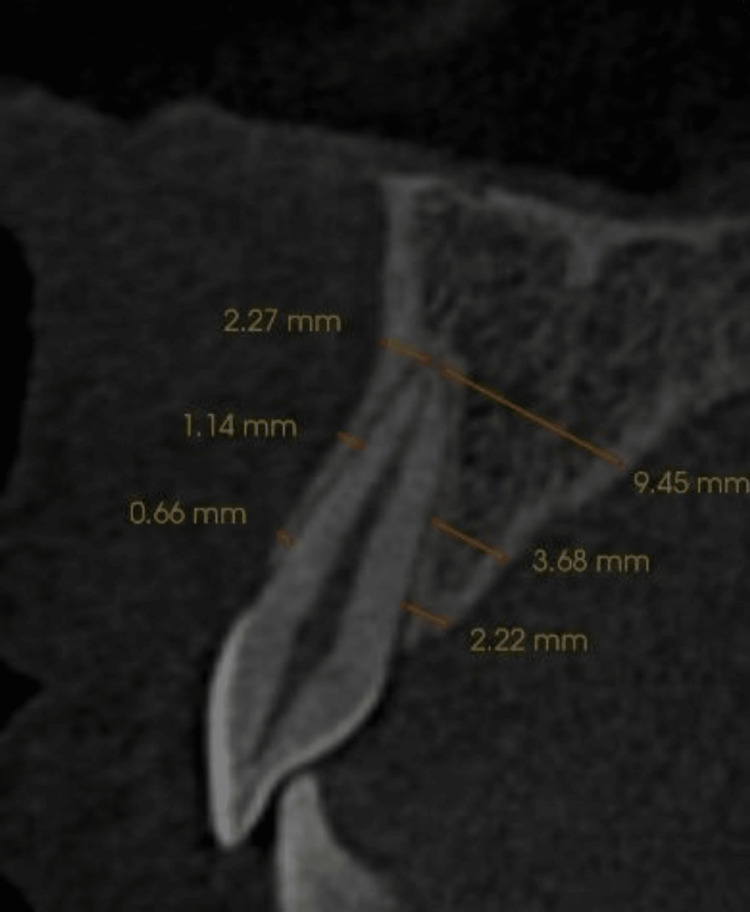
Two orange lines depict the root angulation between the long axis of the root and the long axis of the bone.

The root angulation was measured on a cross-section between the long axis of the root and the long axis of the bone (Figure 6).

## Results

The descriptive statistics were done combined and separately for male and female subjects. The Student’s t test was performed to compare the result for buccal, palatal, mesial, and distal bone thickness around maxillary central and lateral incisors and canines and height from crest to the apex for all subjects (Table 1).

**Table 1:** Descriptive statistics for maxillary central and lateral incisors and canine measurements at different levels at different sites for all subjects at a 5% significance level. CB, canine buccal; CP, canine palatal; CM, canine mesial; CD, canine distal; CA, canine angulation; CH, canine height; LIB, lateral incisor buccal; LIP, lateral incisor palatal; LIM, lateral incisor mesial; LID, lateral incisor distal; LIA, lateral incisor angulation; LIH, lateral incisor height; CIB, central incisor buccal; CIP, central incisor palatal; CIM, central incisor mesial; CID, central incisor distal; CIA, central incisor angulation; CIH, central incisor height L1, at alveolar crest; L2, at mid-root; L3, at apical region

Variables	Mean	SD	95% Lower CI	95% Upper CI
CBL1	1.14	0.59	1.0	1.2
CBL2	0.95	0.61	0.8	1.1
CBL3	1.93	1.29	1.7	2.1
CPL1	2.04	1.76	1.7	2.3
CPL2	3.66	1.45	3.4	3.9
CPL3	7.53	2.9	7.0	8.0
CML1	2.24	0.93	2.1	2.4
CML2	2.56	0.92	2.4	2.7
CML3	7.58	2.18	7.2	7.9
CDL1	2.48	1.39	2.2	2.7
CDL2	2.66	1.06	2.5	2.8
CDL3	6.22	1.93	5.9	6.5
CA	16	8	14.7	17.3
CH	18	4.2	17.3	18.7
LIBL1	1.12	0.6	1.0	1.2
LIBL2	0.96	0.46	0.9	1.0
LIBL3	1.85	1.02	1.7	2.0
LIPL1	1.45	1.01	1.3	1.6
LIPL2	2.65	1.26	2.4	2.9
LIPL3	5.63	2.3	5.2	6.0
LIML1	1.82	0.56	1.7	1.9
LIML2	1.83	0.67	1.7	1.9
LIML3	5.55	1.56	5.3	5.8
LIDL1	2.3	1.1	2.1	2.5
LIDL2	2.56	0.89	2.4	2.7
LIDL3	7.58	2.48	7.2	8.0
LIA	10	8	8.7	11.3
LIH	18.2	3.6	17.6	18.8
CIBL1	1.08	0.45	1.0	1.2
CIBL2	1.06	0.54	1.0	1.1
CIBL3	1.89	1.22	1.7	2.1
CIPL1	1.64	0.72	1.5	1.8
CIPL2	3.18	1.39	2.9	3.4
CIPL3	6.54	2.83	6.1	7.0
CIML1	3	1.19	2.8	3.2
CIML2	2.94	1.12	2.8	3.1
CIML3	7.36	2.28	7.0	7.7
CIDL1	1.83	0.57	1.7	1.9
CIDL2	1.87	0.75	1.7	2.0
CIDL3	5.49	1.57	5.2	5.8
CIA	12	7	10.8	13.2
CIH	18	3.8	17.4	18.6

Descriptives for canine

The mean width of canine buccal L1 in female subjects [1.05 mm, 95% CI (1.0-1.1)] was statistically significantly different from the mean width in male subjects [1.33 mm, 95% CI (1.1-1.5)]. The mean width of canine palatal L2 in female subjects [3.32 mm, 95% CI (3.1-3.6)] was statistically significantly different from the mean width in male subjects [4.4 mm, 95% CI (3.9-4.9)]. The mean width of canine palatal L3 in female subjects [6.99 mm, 95% CI (6.5-7.5)] was statistically significantly different from the mean width in male subjects [8.72 mm, 95% CI (7.8-9.7)]. The mean canine height in female subjects [17.4 mm, 95% CI (16.5-18.3)] was statistically significantly different from the mean width in male subjects [19.4 mm, 95% CI (18.3-20.5)] (Table 2).

**Table 2:** Descriptive statistics for canine measurements at different levels at different sites for both male and female subjects at a 5% significance level. *Statistically significant C, canine; B, buccal; P, palatal; M, mesial; D, distal; A, angulation; H, height L1, at alveolar crest; L2, at mid-root; L3, at apical region

	Female				Male			
Variables	Mean	SD	95% Lower CI	95% Upper CI	Mean	SD	95% Lower CI	95% Upper CI
CBL1*	1.05	0.49	1.0	1.1	1.33	0.73	1.1	1.5
CBL2	0.97	0.67	0.8	1.1	0.9	0.46	0.8	1.0
CBL3	1.97	1.38	1.7	2.2	1.84	1.08	1.5	2.2
CPL1	1.74	1.21	1.5	2.0	2.68	2.48	1.9	3.4
CPL2*	3.32	1.26	3.1	3.6	4.4	1.59	3.9	4.9
CPL3*	6.99	2.56	6.5	7.5	8.72	3.26	7.8	9.7
CML1	2.22	0.94	2.0	2.4	2.29	0.89	2.0	2.6
CML2	2.47	0.89	2.3	2.6	2.76	0.96	2.5	3.0
CML3	7.35	2.04	6.9	7.8	8.1	2.42	7.4	8.8
CDL1	2.61	1.56	2.3	2.9	2.19	0.85	1.9	2.4
CDL2*	2.83	1.12	2.6	3.1	2.3	0.83	2.1	2.5
CDL3	6.27	1.91	5.9	6.7	6.12	1.97	5.5	6.7
CA	15	8	13.4	16.6	17	9	14.3	19.7
CH*	17.4	4.4	16.5	18.3	19.4	3.6	18.3	20.5

Descriptives for lateral incisor 

The mean width of lateral incisor palatal L2 in female subjects [2.31 mm, 95% CI (2.1-2.5)] was statistically significantly different from the mean width in male subjects [3.38 mm, 95% CI (3.0-3.8)]. The mean width of lateral incisor palatal L3 in female subjects [4.97 mm, 95% CI (4.6-5.3)] was statistically significantly different from the mean width in male subjects [7.07 mm, 95% CI (6.3-7.9)]. The mean angulation of lateral incisor in female subjects [9 degrees, 95% CI (7.8-10.2)] was statistically significantly different from the male subjects [14 degrees, 95% CI (11-17)]. The mean lateral incisor height in female subjects [17.6 mm, 95% CI (16.8-18.4)] was statistically significantly different from the male subjects [19.3 mm, 95% CI (18.5-20.1)] (Table 3).

**Table 3:** Descriptive statistics for lateral incisor measurements at different levels at different sites for both male and female subjects at a 5% significance level. *Statistically significant LI, lateral incisor; B, buccal; P, palatal; M, mesial; D, distal; A, angulation; H, height; SD, standard deviation; CI, confidence interval L1, at alveolar crest; L2, at mid-root; L3, at apical region

	Female				Male			
Variables	Mean	SD	95% Lower CI	95% Upper CI	Mean	SD	95% Lower CI	95% Upper CI
LIBL1	1.08	0.49	1.0	1.2	1.22	0.77	1.0	1.4
LIBL2	0.97	0.46	0.9	1.1	0.93	0.46	0.8	1.1
LIBL3	1.88	1	1.7	2.1	1.78	1.08	1.5	2.1
LIPL1	1.35	0.88	1.2	1.5	1.69	1.22	1.3	2.1
LIPL2*	2.31	1.1	2.1	2.5	3.38	1.29	3.0	3.8
LIPL3*	4.97	1.77	4.6	5.3	7.07	2.68	6.3	7.9
LIML1	1.82	0.52	1.7	1.9	1.83	0.63	1.6	2.0
LIML2	1.78	0.54	1.7	1.9	1.94	0.88	1.7	2.2
LIML3	5.43	1.44	5.1	5.7	5.81	1.76	5.3	6.3
LIDL1	2.22	0.94	2.0	2.4	2.47	1.39	2.1	2.9
LIDL2	2.47	0.89	2.3	2.6	2.74	0.88	2.5	3.0
LIDL3	7.35	2.04	6.9	7.8	8.08	3.22	7.1	9.0
LIA*	9	6	7.8	10.2	14	10	11.0	17.0
LIH*	17.6	3.8	16.8	18.4	19.3	2.8	18.5	20.1

Descriptives for central incisor 

The mean width of central incisor palatal L2 in female subjects [2.81 mm, 95% CI (2.6-3)] was statistically significantly different from the mean width in male subjects [4 mm, 95% CI (3.5-4.5)]. The mean width of central incisor palatal L3 in female subjects [6.02 mm, 95% CI (5.5-6.5)] was statistically significantly different from the mean width in male subjects [7.68 mm, 95% CI (6.7-8.7)] (Table 4).

**Table 4:** Descriptive statistics for lateral incisor measurements at different levels at different sites for both male and female subjects at a 5% significance level. *Statistically significant CI, central incisor; B, buccal; P, palatal; M, mesial; D, distal; A, angulation; H, height L1, at alveolar crest; L2, at mid-root; L3, at apical region

	Female				Male			
Variables	Mean	SD	95% Lower CI	95% Upper CI	Mean	SD	95% Lower CI	95% Upper CI
CIBL1	1.04	0.43	1.0	1.1	1.17	0.48	1.0	1.3
CIBL2	1.07	0.49	1.0	1.2	1.04	0.64	0.9	1.2
CIBL3	1.89	1.07	1.7	2.1	1.87	1.51	1.4	2.3
CIPL1	1.55	0.71	1.4	1.7	1.84	0.7	1.6	2.0
CIPL2*	2.81	1.14	2.6	3.0	4	1.54	3.5	4.5
CIPL3*	6.02	2.38	5.5	6.5	7.68	3.4	6.7	8.7
CIML1	3.02	1.3	2.8	3.3	2.97	0.92	2.7	3.2
CIML2	2.82	1.12	2.6	3.0	3.21	1.07	2.9	3.5
CIML3	7.14	2.25	6.7	7.6	7.84	2.3	7.2	8.5
CIDL1	1.82	0.52	1.7	1.9	1.86	0.68	1.7	2.1
CIDL2	1.77	0.52	1.7	1.9	2.08	1.07	1.8	2.4
CIDL3	5.41	1.43	5.1	5.7	5.67	1.85	5.1	6.2
CIA	12	7	10.6	13.4	13	8	10.6	15.4
CIH	17.9	3.9	17.1	18.7	18.3	3.8	17.2	19.4

To detect statistical correlations between measurements of root angulation and buccal cortical thickness, Pearson's correlation two-tailed p values were calculated. The correlation between CBL1 and CA, CBL3 and CA, LIBL3 and LA, and CIBL3 and CA were found to be significant (p < 0.005) (Table 5).

**Table 5:** The correlation of root angulation and cortical thickness at different levels for different teeth. *Statistically significant C, canine; LI, lateral incisor; CI, central incisor; B, buccal L1, at alveolar crest; L2, at mid-root; L3, at apical region

Root angulation plotted against different variables	p-value
CBL1*	0.002
CBL2	0.55
CBL3*	0.001
LIBL1	0.88
LIBL2	0.17
LIBL3*	0.0006
CIBL1	0.4
CIBL2	0.02
CIBL3*	0.003

## Discussion

This study assessed the bone volume around the anterior maxillary teeth using CBCT. The buccal, palatal, mesial, and distal bone thickness was measured around the tooth for immediate implant placement. All the measurements were done at three levels, L1, L2, and L3, corresponding to the crestal region, mid-root region, and apical region of the respective teeth. We also measured available bone height, which affects long-term implant success [8]. Sufficient alveolar ridge height is needed for the success of the implants; vertical bone augmentation is recommended in cases with deficient vertical bone height. Bone augmentation is also required in cases where crestal bone is more than 3-4 mm below the free gingival margin [9]. Root angulation was also measured as it determines sagittal bone thickness. Root angulation outlines the socket as it guides immediate implant placement [10]. Excessively inclined or angulated root reduces the bone thickness along the buccal or the palatal aspect, which may affect bone anchorage and, ultimately, long-term implant success [6]. This technique of measuring the bone volume using CBCT around the potential teeth provides an overall evaluation of the bone volume at the possible implant site.

We observed that the mean thickness of the buccal alveolar bone at the L1 for central incisors, lateral incisors, and canine were (1.08, 1.12, and 1.14 mm) respectively. These values are consistent with the study done by ***AlAli et al. (0.76, 0.79, and 0.83 mm) [11], Othman et al. (0.93, 0.9, 0.95) [12], Sheerah et al. (1.21, 0.93 and 0.88 mm) [13], Han and Jung (0.97, 0.78, and 0.95 mm) [14]. The mean thickness of the buccal alveolar bone at the L2 for central incisors, lateral incisors, and canine were (1.06, 0.96, and 0.95 mm) respectively. These values are consistent with the study by AlAli et al. (1.17, 0.80, and 0.81 mm) [11], Othman et al. (0.81, 0.68, and 0.71 mm) [12], Sheerah et al. (0.96, 0.92, and 0.84 mm) [13], Han and Jung (1.25, 0.85, and 1.42 mm) [14]. The mean thickness of the buccal alveolar bone at the L3 for central incisors, lateral incisors, and canine were (1.89, 1.85, and 1.93 mm) respectively. These values are consistent with the study done by Othman et al. (1.44, 1.36, and 1.34 mm), Sheerah et al. (1.51, 1.58, and 1.38 mm), Han and Jung (1.72, 1.32, and 1.60 mm). Overall the buccal bone thickness of around 1 mm near the crest and mid-root region was observed in the literature. The above values of the alveolar bone thickness showed that the buccal alveolar bone thickness was maximum at the apical region of the alveolus and thinnest at the mid-root region.

The thickness of the buccal wall is very well documented in the literature. However, more information is needed in the literature regarding the significance of the palatal and mesiodistal bone. The palatal bone is critical in immediate implant placement, especially in buccal bone fenestrations and dehiscence [15]. The mean thickness of the palatal alveolar bone at the L1 for central incisors, lateral incisors, and canine were 1.64, 1.45, and 2.04 mm, respectively. These values are consistent with the studies done by AlTarawneh et al. (1.06, 0.87, and 1.10 mm) [16], Han and Jung (0.82, 0.98, and 0.72 mm) [14] and Wang et al. (0.96, 0.81, and 0.78 mm) [17]. The mean thickness of the palatal alveolar bone at the L2 for central incisors, lateral incisors, and canine were (3.18, 3.66, and 3.32 mm) respectively. These values are consistent with the studies done by AlTarawneh et al. (1.66, 1.52, and 1.75 mm), Hanand Jung (3.89, 3.39, and 3.05 mm), and Wang et al. (3.67, 3.16 and 4.11 mm). The mean thickness of the palatal alveolar bone at the L3 for central incisors, lateral incisors, and canine were (6.54, 5.63, and 7.53 mm) respectively. This data is consistent with AlTarawneh et al. (3.13, 2.66, and 3.60), Han and Jung (5.9, 4.9, and 4.7 mm), and Wang et al. (7.57, 6.57, and 8.79 mm). The above values of the alveolar bone thickness showed that the palatal alveolar bone is much thicker around the roots of all three anterior teeth; this data appears consistent with the literature where the palatal bone is thicker than the buccal bone [6, 16]. The thickness was maximum at the apical region of the alveolus, whereas it was thinnest at the crestal region. Comparing the buccal and palatal measurements of the alveolus, it was observed that the buccal bone thickness is less compared to the palatal bone, primarily due to the root angulation in the sagittal plane [6].

The mesial and distal bone thickness must also be considered in implant planning. It helps align the implant in the arch without violating the proximity to the adjacent teeth, especially in cases with periapical infections associated with the adjacent tooth. The areas with periapical infections or inflammatory changes are unfavorable for achieving successful osseointegration [18]. There are no documented studies in the literature measuring mesial and distal bone thickness. 

The angles between the long axis of the tooth and the alveolar bone for central incisors, lateral incisors, and canine were (12°, 10°, and 16°) respectively. The measurements were significantly different than Zhang et al. (17.65°, 18.79°, and 23.82°) study [19]. The difference between these measurements could be because of the population difference; the above two studies were done in the Chinese population. We measured alveolar bone height from the crest to the proximal vital anatomical structure. We found that the available bone height for central incisors, lateral incisors, and canine was (18, 18.2, and 18 mm), consistent with the study by Zhang et al. (18.83, 19.07, and 18.91 mm) [20].

The results were compared between male and female subjects. Canine palatal, distal, and height measurements were significantly high in male subjects. Similarly, lateral and central incisor palatal measurements and lateral incisor angulation and height were significantly higher in males. These results were consistent with the study done by AlTarawneh et al. [16]. The study performed by Wang et al. [17] documented the statistically significant difference between males and females. 

Limitations of our study included that we only included scans with good resolution and limited artifacts; however, in typical situations, we would encounter poor quality scans with several inherent artifacts, which limits the evaluation of fine details and may also limit the assessment of bone thickness. The CBCT is an ideal imaging modality for the pre-surgical measurements before immediate implant placement; however, the study by Alsino et al. [21], reported that CBCT overestimates the measurements as compared to the actual measurements. 

## Conclusions

The results of the study are consistent with the literature. We observed that the labial bone thickness is generally thin, with approximate measurements around 1 mm; it was also observed that it was the narrowest in the mid-root region of lateral incisors. This affects the immediate implant placement and soft/hard tissue augmentation in the anterior maxillary area. The other result of the study suggested that the canine was the most angulated tooth with a more buccal alveolar bone thickness, which is better for immediate implant placement. The study was conducted on CBCT images and CBCT was found to be a reliable modality for the pre-surgical immediate implant planning. 
